# *QuickStats*: Emergency Department Visit Rates,*^,^^†^ by Age Group — United States, 2019–2020

**DOI:** 10.15585/mmwr.mm7142a5

**Published:** 2022-10-21

**Figure:**
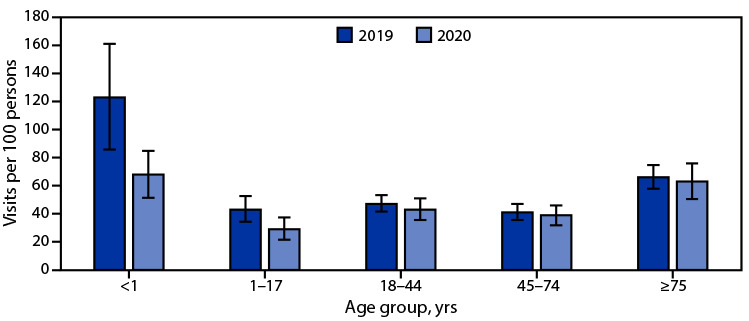
The emergency department (ED) visit rate for infants aged <1 year declined by nearly one half from 123 visits per 100 infants during 2019 to 68 during 2020. The ED visit rate for children and adolescents aged 1–17 years also decreased from 43 to 29 visits per 100 persons during the same period. Decreases among adults aged 18–44 (47 to 43 per 100 adults), 45–74 (41 to 39), and ≥75 years (66 to 63) from 2019 to 2020 were not statistically significant. ED visit rates were highest for infants aged <1 year followed by adults aged ≥75 years.

**Source**: National Center for Health Statistics, National Hospital Ambulatory Medical Care Survey, 2019–2020.

